# Learning generalizable behaviors from demonstration

**DOI:** 10.3389/fnbot.2022.932652

**Published:** 2022-10-03

**Authors:** Corban Rivera, Katie M. Popek, Chace Ashcraft, Edward W. Staley, Kapil D. Katyal, Bart L. Paulhamus

**Affiliations:** Johns Hopkins Applied Physics Laboratory, Intelligent Systems Center, Laurel, MD, United States

**Keywords:** neural networks, robotics, artificial intelligence, temporal network, learning from demonstration

## Abstract

Generalizing prior experiences to complete new tasks is a challenging and unsolved problem in robotics. In this work, we explore a novel framework for control of complex systems called Primitive Imitation for Control (*PICO*). The approach combines ideas from imitation learning, task decomposition, and novel task sequencing to generalize from demonstrations to new behaviors. Demonstrations are automatically decomposed into existing or missing sub-behaviors which allows the framework to identify novel behaviors while not duplicating existing behaviors. Generalization to new tasks is achieved through dynamic blending of behavior primitives. We evaluated the approach using demonstrations from two different robotic platforms. The experimental results show that *PICO* is able to detect the presence of a novel behavior primitive and build the missing control policy.

## 1. Introduction

Neural networks have demonstrated extraordinary ability to control systems with high degrees of freedom. An important challenge is how to control such high-degree of freedom systems with only a few control inputs. One approach to address the scaling challenge is through modularity and hierarchical control mechanisms (Akinola et al., 2017; Shazeer et al., 2017; Zhao et al., 2017). These approaches use the limited number of inputs to select a primitive control policy, from a library of primitive behaviors, and potentially a target. Complex tasks are performed by chaining primitive behaviors.

**Figure 1:**
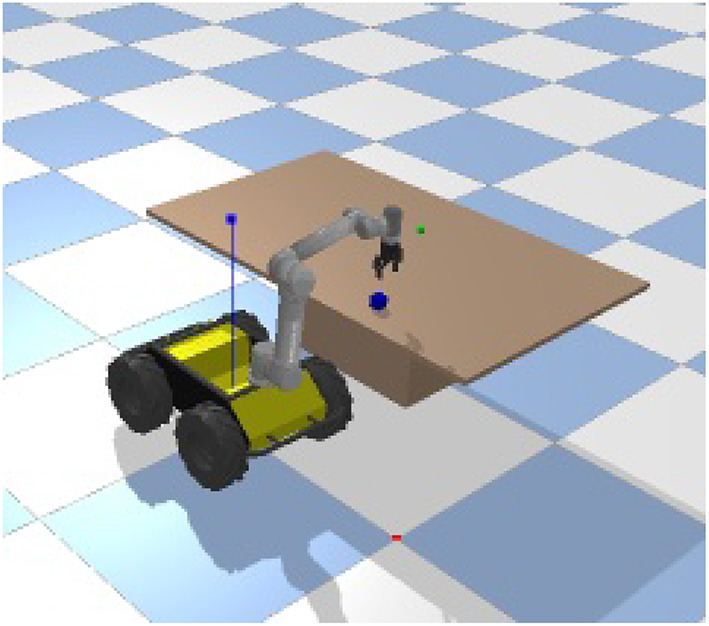
Husky-UR5 reach and grasp environment.

As an example of this scenario, consider a Universal Robots UR5 (UR, 2019) manipulator mounted on a Clearpath Husky platform (Hus, 2019) as shown in Figure 1. The UR5 is used to demonstrate reaching, grabbing, and lifting a block on a table. Other tasks may require performing these actions in another order, so it may be useful to learn and maintain a collection of these primitive behaviors for later use. While the underlying behavior primitives are well-defined for the reach-and-grasp scenario, other example scenarios may not have as well-defined or labeled primitives. In this work, we assume that the underlying label of the behaviors shown in the task demonstrations is unknown.

The questions we investigate are how might we learn and maintain the primitive library from unlabeled demonstrations and, assuming the behavior primitive library exists, how would one know when to use, adapt, or create a new primitive behavior. We propose that the behavior library should be actively maintained to minimize redundancy and maximize the ability to reconstruct complex tasks through chains of the primitive behaviors. In this work, we explore techniques to directly optimize for these criteria by building on methods that learn from demonstration.

We explore maintaining a behavior primitive library in an online learning scenario. Given a potentially non-empty behavior primitive library and a new set of unlabeled task demonstrations, we seek to update the behavior primitive library to maximally accommodate the new demonstrations while maintaining the ability to reconstruct previously demonstrated trajectories.

**Figure 2:**
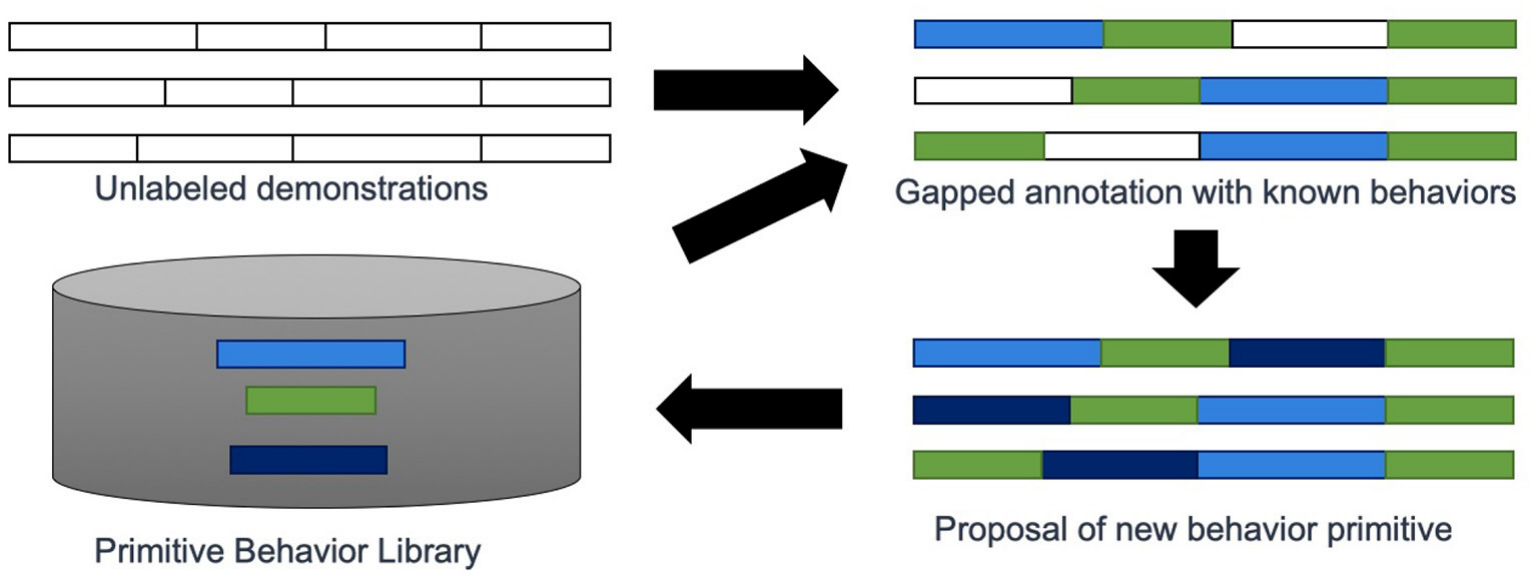
An overview of *PICO*. The approach takes as input unlabeled demonstrations and a library of primitive behaviors. The goal is to predict the primitive behavior label associated with each time point in all demonstrations. Additional behavior primitive models can be trained to fill gaps that are not well-represented by existing behavior primitives.

Our contribution is an approach called *PICO* that simultaneously learns subtask decomposition from unlabeled task demonstrations, trains behavior primitives, and learns a hierarchical control mechanism that allows blending of primitive behaviors to create even greater behavioral diversity, an overview is shown in Figure 2. Our approach directly optimizes the contents of the primitive library to maximize the ability to reconstruct unlabeled task demonstrations from sequences of primitive behaviors.

## 2. Preliminaries

Learning from demonstration (LfD) and imitation learning allow agents to execute a task by observing the task being performed (Hussein et al., 2017). In the robotics domain, a goal of imitation learning is to produce a mapping, π, from states to actions, known as a control *policy* (Argall et al., 2009; Schaal and Atkeson, 2010), that has the maximum likelihood of producing the demonstration dataset $D=\{\rho_{1},\rho_{2},\ldots ,\rho_{n}\}$, where each ρ = (*s*_1_, *a*_1_), (*s*_2_, *a*_2_), …, (*s*_*T*_, *a*_*T*_) is a demonstration trajectory of state, action pairs. The demonstrations can be created by another control policy (Rusu et al., 2015), by a human expert (Konidaris et al., 2012), or in a simulated environment (Shiarlis et al., 2018; Kipf et al., 2019). Let π_θ_ parameterized by θ. The goal is then to optimize Equation (1) by varying θ.


(1) $$\begin{array}{l} \max E_{\rho }\left[\overset{T}{\underset{t=1}{\sum }}\log\pi_{\theta }\left(a_{t}|s_{t}\right)\right] \end{array}$$

Following optimization, covariate drift can cause errors in the control process that can place the robot in a previously unobserved state. Control policies will have higher action prediction errors in parts of the state space that it has not observed, leading to poor action predictions and compounding errors with increased iterations of the policy. One approach that has been introduced to decrease the impact of covariate shift is to introduce noise into the demonstrations used for learning (Laskey et al., 2017). This approach increases the amount of state space covered by the policy and improves action predictions around the demonstrations, leading to better generalization and error tolerance.

### 2.1. Model agnostic meta-learning

In meta-learning a model is trained on a variety of learning tasks and the parameters of the method are fine-tuned for generalization. The idea of meta-learning is to combine a set of learner models to improve performance on a task more quickly than one without pretrained models. This is a common strategy for one-shot (Santoro et al., 2016) or few shot scenarios, where a model must be trained using one or a few examples. Some approaches for meta-learning come from the reinforcement learning (Finn et al., 2017), which typically differ in how they update individual learners. Some meta-learning methods update models using gradient information (Finn et al., 2017) and others learn how to update learners from data (Bengio et al., 2002; Andrychowicz et al., 2016).

## 3. Related work

Imitation learning alone does not provide a mechanism to generalize demonstrations to new tasks. One mechanism to address this challenge is task decomposition, which has the goal of identifying subtasks from demonstration. Subtasks can be made into sub-policies through imitation learning, including methods that combine subtask discovery with imitation learning (Shiarlis et al., 2018; Xu et al., 2018). By decomposing demonstrations into subtasks, it becomes possible to permute the sequence of sub-policies to achieve greater task diversity and generalizability. However, decomposing demonstrations into subtasks that are maximally useful for recombination is a challenge in task decomposition (Shiarlis et al., 2018).

Once sub-task policies are established, a hierarchical control policy can be learned that identifies the sequence of policies needed to achieve a specified goal. Given a sufficiently diverse set of demonstrations the reasoning layer can be learned from a set of demonstrations (Xu et al., 2018). Several approaches for learning hierarchical architectures for control policies from limited demonstrations have been proposed (Duan et al., 2017; Shiarlis et al., 2018; Xu et al., 2018). We were inspired by the work on mixtures-of-experts (Jacobs et al., 1991; Shazeer et al., 2017) which includes a similar hierarchical representation.

Some approaches assume that the behavior primitive library is fully trained in advance (Xu et al., 2018). In the reinforcement learning domain, the options framework (Stolle and Precup, 2002; Kulkarni et al., 2016; Andreas et al., 2017), and hierarchical reinforcement learning (Dietterich, 2000) are common approaches for organizing hierarchies of policies. The techniques in reinforcement learning are often predicated on being able to interact with an environment and collect a lot of data. In this work, we focus on learning hierarchical task decomposition strategies from a limited set of demonstrations.

### 3.1. Task sketch for sub-policy discovery

Some related approaches (Andreas et al., 2017; Mu et al., 2019) perform demonstration decomposition by combining both demonstrations and task sketches. The literature refers to these approaches as *weakly-supervised* because the order of tasks is given and the exact transition points within a demonstration must be inferred.

Let D be our dataset containing trajectories ρ = [(*s*_0_, *a*_0_), (*s*_1_, *a*_1_), …, (*s*_*T*_, *a*_*T*_)] of length *T* containing state-action tuples (*s, a*) for state *s* and action *a*. Given a library of sub-tasks policies $B=\left(\pi_{1},\pi_{2},\ldots ,\pi_{K}\right)$, A task sketch τ = (τ_1_, τ_2_, …, τ_*L*_) is a sequence of sub-tasks labels where *L* is the length of the sketch. A path is a sequence of sub-task labels ζ = (ζ_1_, ζ_2_, …, ζ_*T*_) where *T* is the length of a demonstration. We assume that *L* < < *T*. We say that a path ζ matches a task sketch τ if τ = ζ after removing all adjacent duplicate sub-tasks in ζ. For example, the path (π_2_, π_2_, π_2_, π_3_, π_3_, π_1_, π_1_, π_1_, π_1_) matches the task sketch (π_2_, π_3_, π_1_).

## 4. Methods

In this section, we describe the approaches most closely aligned with our work referred to as CTC (Graves et al., 2006) and TACO (Shiarlis et al., 2018). Then, we introduce our approach called Primitive Imitation for Control (*PICO*).

### 4.1. Connectionist temporal classification

Given a dataset D and task sketch τ, one approach to obtain a set of generalizable sub-tasks B is to separately learn alignment of trajectories to the task sketch then learn the control policies for sub-tasks with behavior cloning. Connectionist Temporal Classification (CTC) (Graves et al., 2006) addresses the problem of aligning sequences of dissimilar lengths. There are potentially multiple ways in which a path could be aligned to a task sketch. Let ℤ_(*T*, τ)_ be the set of all paths of length *T* that match the task sketch τ. The CTC objective maximizes the probability of the task sketch τ given the input trajectory ρ:


(2) $$\begin{array}{l} \theta^{*}=\underset{\theta }{\text{argmax}}E_{\left(\rho ,\tau \right)}\left[p_{\theta }\left(\tau |\rho \right)\right] \end{array}$$

(3) $$\begin{array}{l} \theta^{*}=\underset{\theta }{\text{argmax}}E_{\left(\rho ,\tau \right)}\left[\underset{\zeta \in {\mathbb{Z}}_{\left(T,\tau \right)}}{\sum }\overset{T}{\underset{t=1}{\prod }}p_{\theta }\left(\zeta_{t},|\rho_{t}\right)\right] \end{array}$$

*p*_θ_(ζ_*t*_, |ρ) is commonly represented as a neural network with parameters θ that outputs the probability of each sub-task policy in B. The objective is solved efficiently using dynamic programming. Inference using the neural network model is used to find a maximum likelihood path ζ for a trajectory ρ. The labels in ζ provide an association between state-action tuples (*s*_*t*_, *a*_*t*_) and subtask policies π∈B. The state-action policies associated with a single sub-task are used to create a sub-task policy using behavior cloning.

### 4.2. Temporal alignment for control

Given a demonstration ρ and a task sketch τ, Temporal Alignment for Control (TACO) (Shiarlis et al., 2018) will learn where each subtask begins and ends in the trajectory and simultaneously trains a library of sub-tasks policies B. TACO maximizes the joint log likelihood of the task sequence τ and the actions from sub-task policies contained in B conditioned on the states. Let **a**_ρ_ and **s**_ρ_ be the set of actions and states, respectively in trajectory ρ.


(4) $$p(\tau ,a_{\rho }|s_{\rho })=\sum_{\zeta \in {\mathbb{Z}}_{\left(T,\tau \right)}}p(\zeta |s_{\rho })\prod_{t=1}^{T}\pi_{\zeta_{t}}(a_{t}|s_{t})$$

where *p*(ζ|**s**_ρ_) is the product of action probabilities associated with any given path ζ. The path ζ determines which data within ρ corresponds to each sub-task policy π and $\prod_{t=1}^{T}\pi_{\zeta_{t}}\left(a_{t}|s_{t}\right)$ is the behavior cloning objective from Equation (1).

### 4.3. Primitive imitation for control (PICO)

In this work, we introduce Primitive Imitation for Control (*PICO*). The approach differs from previous work in a few important ways. *PICO* similarly decomposes behavior primitives from demonstration. It optimizes the action conditioned on state and does not require a task sketch, and unlike CTC (Graves et al., 2006), our approach simultaneously learns to segment demonstrations and trains underlying behavior primitive models.

We aim to reconstruct the given trajectories as well as possible using the existing sub-task policy library. As shown in Equation (5), we seek to minimize the sum of squared error between the observed action and the predicted action for all actions over all timepoints *T* and all trajectories ρ∈D. We refer to this objective as minimizing reconstruction error. Let (*s*_ρ_*t*__, *a*_ρ_*t*__) be the state-action tuple corresponding to ρ_*t*_ timepoint *t* in trajectory ρ. The action prediction, Equation (6), is the product of the probability *p*(π|*s*_ρ_*t*__) of a sub-task policy π conditioned on the state *s*_ρ_*t*__ and the action predicted by policy π(*s*_ρ_*t*__) for the state *s*_ρ_*t*__. Substituting Equation (6) into Equation (5) results in Equation (7) which is the optimization problem for *PICO*.


(5) $$\min\sum_{\rho \in D}\sum_{t=0}^{T}{\left(a_{\rho_{t}}-{\hat{a}}_{\rho_{t}}\right)}^{2}$$

(6) $${\hat{a}}_{\rho_{t}}=\sum_{\pi \in ℬ}p(\pi |s_{\rho_{t}})\pi (s_{\rho_{t}})$$

(7) $$\min\sum_{\rho \in D}\sum_{t=0}^{T}[a_{\rho_{t}}-\sum_{\pi \in ℬ}p(\pi |s_{\rho_{t}})\pi (s_{\rho_{t}}){]}^{2}$$

### 4.4. Neural network architecture

Estimates of both *p*(π|*s*_ρ_*t*__) and π(*s*_ρ_*t*__) are given by a recurrent neural network architecture. **Figure 4** gives an overview of the recurrent and hierarchical network architecture. We solve for the objective in Equation (7) directly by back propagation through a recurrent neural network with Equation (5) as the loss function. The model architecture is composed of two branches that are recombined to compute the action prediction at each timepoint.

To more easily compare with other approaches that do not blend sub-task policies, we estimate the maximum likelihood sub-task policy label at each timepoint. We refer to sub-task policies as behavior primitives. The behavior primitive label prediction is given by the maximum likelihood estimate of π shown in Equation (8) for time *t* in trajectory ρ.


(8) $$\underset{{}_{\pi \in ℬ}}{argmax\;}p(\pi |\rho_{t})$$

**Figure 3:**
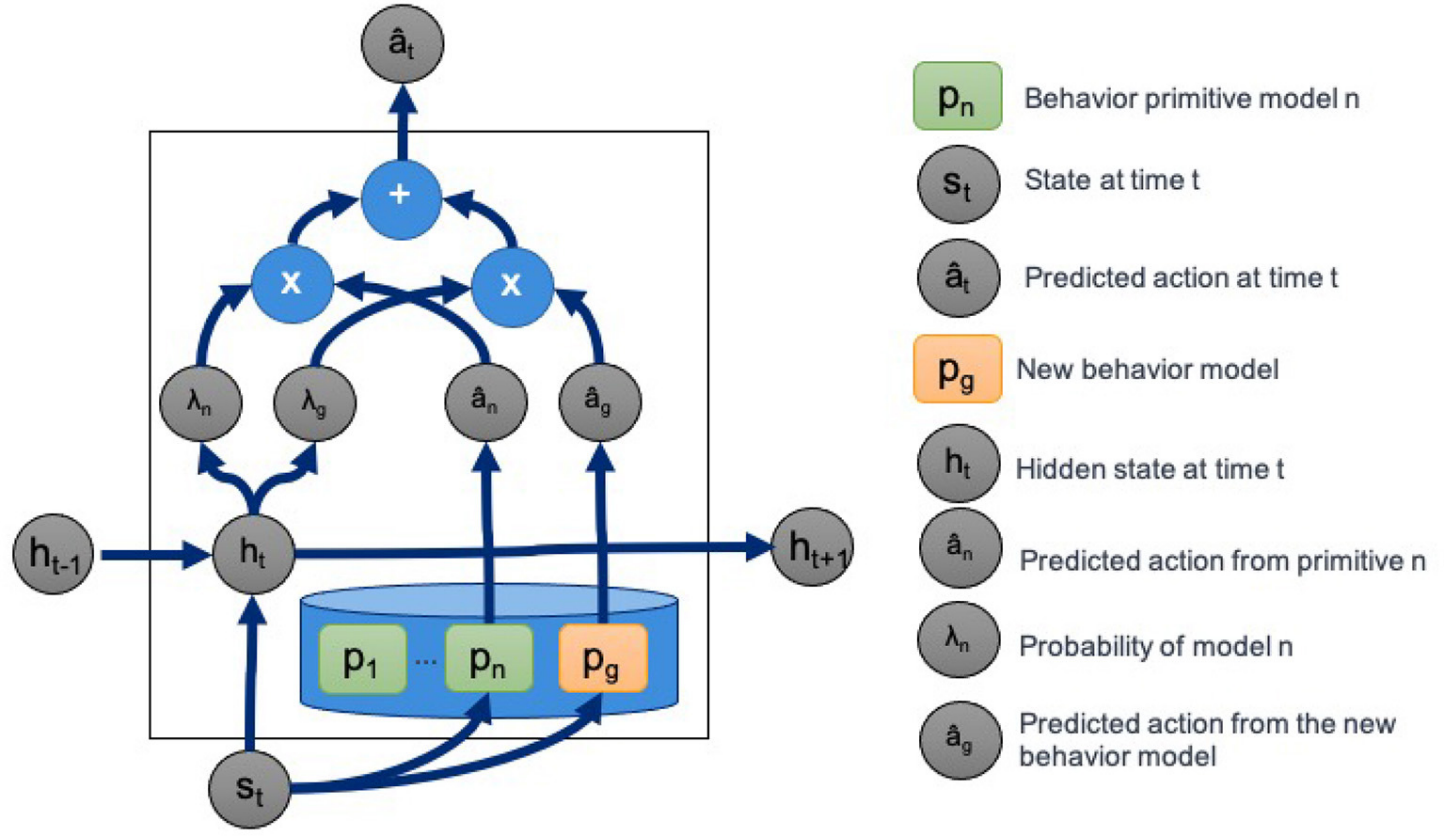
Hierarchical recurrent deep network architecture for task decomposition, novel behavior primitive discovery, and behavior blending.

Figure 3 illustrates how we compute the predicted action â_*t*_ at time *t*. In the figure, the probability of π given state *s*_ρ_*t*__ is λ_π_ = *p*(π|*s*_ρ_*t*__) for π∈B. The latent representation *h*_*t*_ at the current timepoint *t* is a function of both the value of the latent representation of the previous state *h*_*t*−1_ and the current state *s*_*t*_.

**Figure 4:**
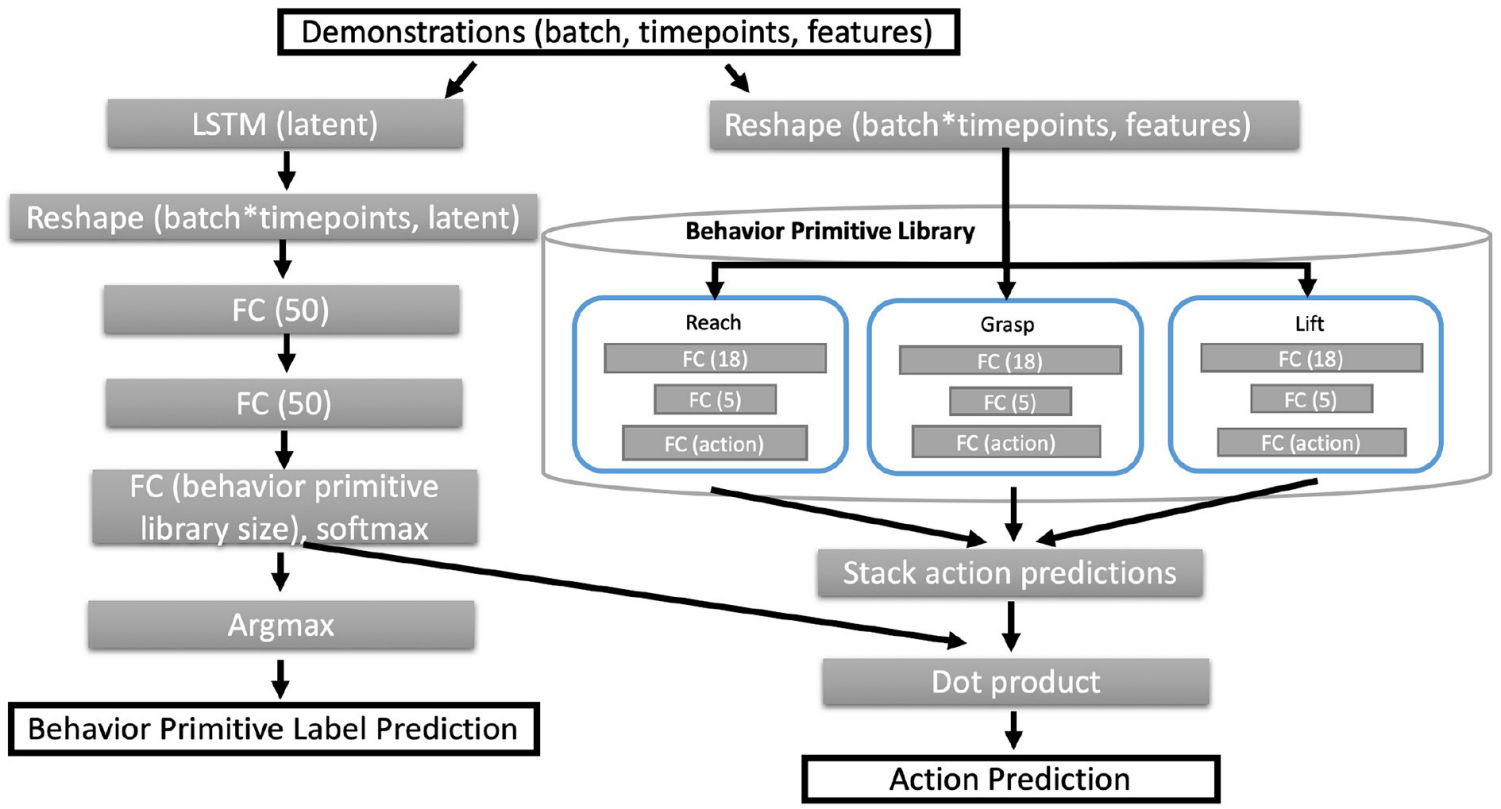
Neural network architecture for *PICO*. Given a set of input trajectories and a behavior primitive library, the core architecture follows two branches, the left most branch estimates a distribution over the behavior primitives. The right hand branch estimates the action prediction from each primitive behavior sub-model. We compute the predicted action as a linear combination between the behavior primitive distribution and the set of predicted actions from all behavior primitives.

Figure 4 details the architecture used for *PICO* based on the Husky+UR5 dataset example. Unless otherwise specified, the fully connected (FC) layers have ReLU activations, except for the output layers from behavior primitive models. The last layer of behavior primitive models have linear activations to support diverse action predictions. While not shown in Figure 4, the network architecture also returns the predicted latent embedding and behavior primitive distribution for additional visualization and analysis.

### 4.5. Discovering and training new behavior primitives

An important aspect of our approach is the ability to discover and create new behavior primitives from a set of trajectories and a partial behavior primitive library. *PICO* detects and trains new behavior primitive models simultaneously. As shown in Figure 3, *PICO* supports building new behavior primitive models by adding additional randomly initialized behavior models to the library prior to training. For our experiments, we assume that we know the correct number of missing primitives.

We define a *gap* in a trajectory as region within a demonstration where actions are not predicted with high probability using the existing behavior primitive models. A gap in a trajectory implies that the current library of behavior primitives is insufficient to describe a set of state-action tuples ρ in some part of the given trajectory. This also implies that the probability *p*(π|ρ_*t*_) that the data ρ_*t*_ for time point *t* was generated by the current library of behavior primitive models is low for all π∈B. These low probabilities increase the likelihood that an additional randomly initialized behavior primitive policy π_*new*_ might have a higher probability *p*(π_*new*_|ρ_*t*_)>*p*(π|ρ_*t*_) for π∈B. The data ρ_*t*_ is then used to train π_*new*_. For nearby data in the same gap region ρ_*t*+1_, it is now more likely that *p*(π_*new*_|ρ_*t*+1_)>*p*(π|ρ_*t*+1_) for π∈B. This mechanism allows π_*new*_ to develop in to a new behavior primitive that is not well-covered by existing primitives.

### 4.6. Training details

*PICO* is trained end-to-end by back propagation. This is possible because all functions in the model are differentiable with the exception the argmax function. For experiments making use of pretrained behavior primitive models, the contents of the behavior primitive library are trained using the DART (Laskey et al., 2017) technique for imitation learning.

As shown in Equation (5), the loss used to train the model is mean squared error between the predicted and observed actions over all timepoints and all demonstrations. There is no loss term for label prediction accuracy, because we assume that the demonstrations are unlabeled.

### 4.7. Metrics

Two metrics are computed to estimate performance. First, we evaluate mean squared error (MSE) as shown in Equation (5) between the predicted and given action. Second, we compute behavior primitive label accuracy which is a comparison between the predicted and given behavior primitive label. Label accuracy is computed as the number of matching labels divided by the total number of comparisons. Both metrics are computed over all timepoints and over all demonstrations in the test set.

### 4.8. Baseline implementations

Shiarlis et al. (2018) developed TACO, which aligned subtasks to demonstrations given a library of primitives and a *task sketch*, where a task sketch describes the sequence in which subtasks will appear. In addition, in their recent work (Shiarlis et al., 2018), they extended the connectionist temporal classification (CTC) algorithm (Graves et al., 2006), commonly used to align sequences for speech recognition, for use with identifying subtasks. For this work, we use TACO and the extended version of CTC as baseline comparisons for our algorithm, using an open source implementation^1^. Both were tested using MLP and RNN architectures.

## 5. Experiments and discussion

We evaluate *PICO* using a reach-grab-lift task using a Husky+UR5 environment. The dataset consists of 100 demonstrations of a Clearpath Husky robot with a UR5 manipulator performing a variety of reach, grasp, and lift tasks (see Figure 1). The number of time steps in the demonstrations varied from 1,000 to 1,800, but each used all three primitives: reach, grasp, and lift.

The first experiment quantifies the ability of *PICO* to identify primitive task labels from demonstration independently from learning behavior primitives. The second experiment evaluates the ability of *PICO* to identify parts of demonstrations that are not represented by existing behavior primitives and rebuild the missing behavior primitive.

### 5.1. Reconstruction from existing primitives

Our initial experiment is an ablation study that separately evaluates the estimate of the primitive behavior probability distribution and the action predictions from learning behavior primitives. We train and freeze behavior primitive models for *reach, grasp*, and *lift* using the ground truth labeled data from trajectories. We evaluated *PICO*, TACO (Shiarlis et al., 2018), and CTC based on label classification accuracy. For Taco and CTC we additionally compared the methods using MLP and RNN based underlying network models. We evaluated all methods based on an 80/20 split of demonstrations into training and test sets. The average of five independent runs were obtained for each approach. In Table 1, we show the results of the comparison.

**Table 1:** Method comparisons using the Husky UR5 Reach and Grasp dataset.

**Husky UR5**	**Label accuracy (%)**	**MSE action prediction**
*PICO*	**96**	**0.053**
TACO (MLP)	74	3.59
TACO (RNN)	73	3.75
CTC (MLP)	25	4.20
CTC (RNN)	33	2.68

The bold values indicate the highest scores observed in each category.

**Figure 5:**
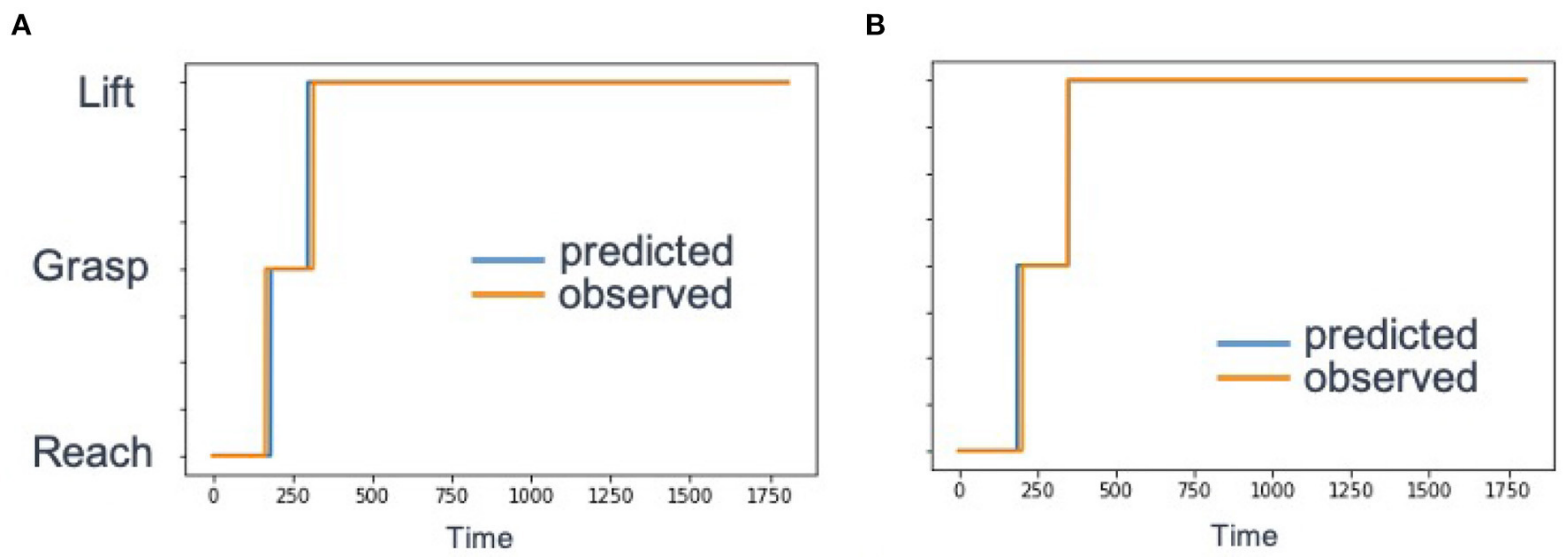
Example behavior primitive label accuracy for a single test demonstration. We compared the label predictions given by *PICO* (red) to the ground truth (blue). **(A)** A sample reconstruction for a single trajectory with an existing behavior primitive library. Timepoints are on the x-axis. and behavior primitive label is on they y-axis. The labels 0, 1, and 2 correspond to reach, grasp, and lift, respectively. **(B)** Reconstruction of an example trajectory and discovery of a missing behavior primitive (grasp).

Figure 5A shows a comparisons between the predicted label based on Equation (8) and the ground truth label. Over all trajectories in the test set, the average label classification accuracy was 96% compared to the ground truth label. The summary of results are shown in Table 1.

### 5.2. Behavior primitive discovery

**Figure 6:**
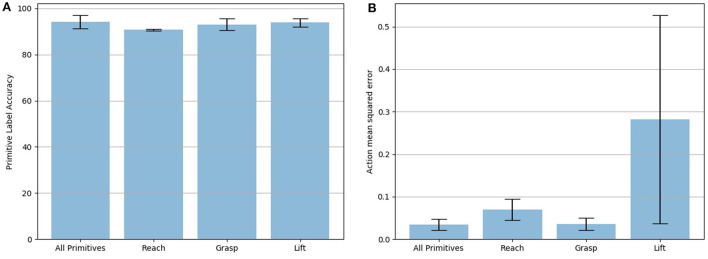
Accuracy of *PICO* to correctly identify a primitive's label on the validation set (20 randomly selected trajectories). **(A)** The leftmost bar shows performance when all primitives are in the library, successive bars denote accuracy when the *reach, grasp*, and *lift* primitives are dropped out and learned from a randomly generated “gap” primitive. Error bars represent the standard deviation across five validation trials. **(B)** Mean squared error between the ground truth action and the learned model's estimate averaged across 20 randomly selected test trajectories five times.

In our next experiment, we evaluate the ability of *PICO* to recognize and build a missing behavior primitive model. We ran a leave-one-behavior-out experiment where one of the three primitives (i.e., reach, grasp, lift) was replaced with a randomly-initialized behavior primitive. This experiment used the same 100 trajectories on the Husky+UR5 dataset discussed in the previous section and a 80/20 split between training and validation sets. Again, five trials were run with the training and validation sets randomly chosen. The label accuracy and action prediction MSE are shown in Figure 6. The leftmost bar shows the results with all primitives pre-trained with behavior cloning. The remaining bars show the accuracy when reach, grasp, and lift, respectively, were replaced with the gap primitive. Note, the gap primitive was updated throughout the training with back-propagation such that the final primitive ideally would perform as well as the original pre-trained, behavior-cloned version; this comparison is shown with the action prediction MSE. The error bars show the standard deviation across the five trials. While the label accuracy across all three replaced primitives is approximately the same, the action prediction for the lift primitive is significantly worse. We believe this is due to the larger variance in lift trajectories. Unlike the reach and grasp which have restrictions placed on their final target position (it needs to be near the block), the final position of lift is randomly placed above the block's starting position.

As shown in the sample trajectory in Figure 5B, the label prediction of the trained model closely aligns with the ground truth label from the example trajectory. Over all of the test trajectories, the average label classification accuracy was 96%.

### 5.3. Visualizing the learned latent space

**Figure 7:**
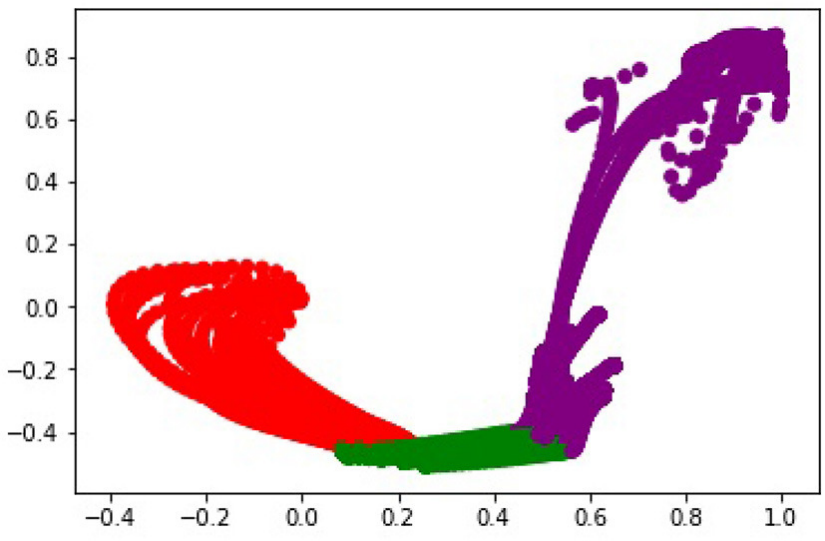
The organization of the learned latent space associated with the Husky-UR5 dataset for reach, grasp, and lift (red, green, and purple, respectively).

To better understand the role of the embedding space for predicting the primitive probability distribution, we visualized the embedding of all states vectors from the test set in the recurrent hidden layer. We would expect that a useful latent embedding would naturally cluster states that correspond to different primitives into distinct locations in the embedding space. Figure 7 shows layout of the latent space in two dimensions. Each point corresponds to a state vector from the test dataset. The points are colored by the ground truth label.

### 5.4. Jaco dial domain dataset

**Figure 8:**
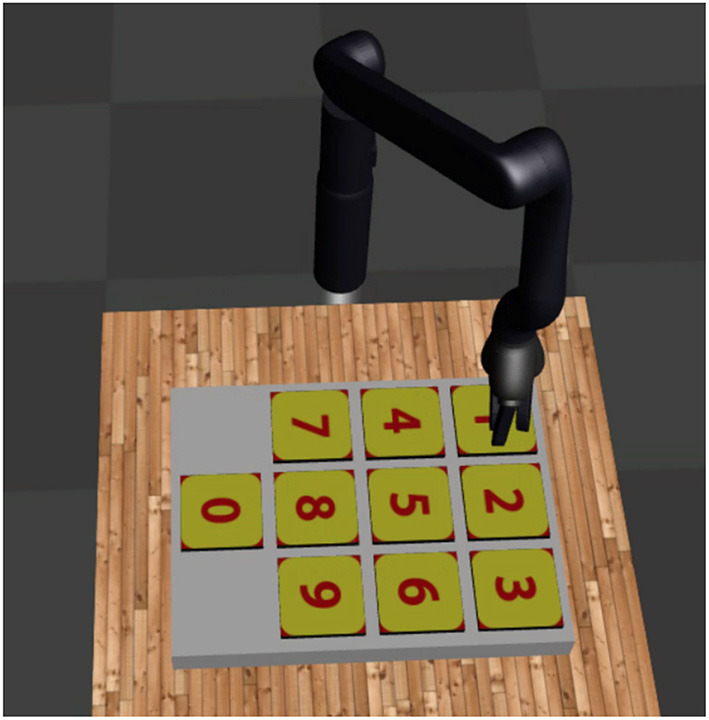
The joint-domain dial scenario. A Jaco manipulator modeled in Mujoco presses a sequences of four keys on a dialpad. The positions of the keys are randomly shuffled for each demonstration. The positions of the joints and positions of the keys are given as state information.

We also make use of the Jaco dial domain dataset (Shiarlis et al., 2018) illustrated in Figure 8. The dial dataset is composed of demonstrations from a Jaco manipulator pressing four keys in sequence (e.g., 3, 5, 4, 5). The positions of the keys are randomly shuffled for each demonstration, but the position of each key is given in the state vector. Repetitive use of primitives are allowed in the demonstrations. The intention with this dataset is to treat pressing an individual digit as a behavior primitive. For this dataset, label prediction accuracy is a challenging metric without a task sketch because the starting position of the Jaco may not provide clues about which button will be pressed. As the Jaco gets closer to a button, it becomes more clear which button will be pressed. The dataset of dialpad demonstrations were generated using default parameters and code from TACO (Shiarlis et al., 2018).

### 5.5. Dial domain comparison

The goal of this comparison is to evaluate the label prediction accuracy of the metacontroller in *PICO*. To isolate the label predictions of the metacontroller, the behavior primitive library is pretrained on the training dataset including 1,200 demonstrations and frozen. Label classification and action prediction accuracy is then evaluated on the test set including 280 demonstrations.

The average results of five runs are shown for TACO and CTC. We evaluate each approach using the same label accuracy and action prediction metrics. The summary of results are shown in Table 2. We found that our approach achieves the highest label accuracy at 65%. The overall label accuracy of *PICO* on the dial dataset is lower than the Husky+UR5 dataset. Additional analysis revealed that many of the mislabeling occurred at the beginning of a new key press where context about where the Jaco is moving next is weakest. The dataset is also more challenging than the Husky dataset because the number of unique behavior primitives has increased from 3 to 10.

**Table 2:** Method comparisons using the Jaco Pinpad dataset.

**Jaco Pinpad**	**Label accuracy**	**MSE action prediction**
*PICO*	**65%**	**0.0061**
TACO (MLP)	47%	0.55
TACO (RNN)	*	*
CTC (MLP)	31%	0.57
CTC (RNN)	29%	0.58

*TACO (RNN) resulted in NaN loss after repeated attempts.

The bold values indicate the highest scores observed in each category.

Also of note, we compare our results to TACO which is a weakly supervised approach. TACO is given the ordering of tasks. For task sequences of length 4, this means that a random baseline would be expected to achieve an accuracy of 25%. For an unlabeled approach like *PICO*, any of the 10 behavior primitives could be selected at each timepoint. With only unlabeled trajectories, the expected accuracy of a random baseline would be 10%.

In both the reach and grasp and pinpad domain, we observed large performance gains in our approach over both TACO and CTC. Both and TACO align actions to behaviors and train new behaviors in a single end-to-end model. In contrast, CTC optimizes the alignment of actions to behavior labels separately from training new primitive behaviors. Shiarlis et al. (2018) point out that the joint optimization results in superior performance of TACO over CTC. We can partially attribute the superior performance of our approach over CTC to the use of an end-to-end model and joint optimization.

We also observed superior performance of our approach over TACO (Shiarlis et al., 2018). The key difference between our approach and TACO is that our approach uses soft attention over behavior primitives to learn a behavior blending strategy. In contrast, TACO makes use of hard attention over behaviors for action prediction. Consequently, our approach effectively makes use of more parameters for each action prediction increasing the expressive power of our approach relative to TACO with a similar network size.

Several interesting open challenges remain. The introduction of methods that can accommodate any number of behaviors without reshaping the core network would be a valuable next step. Also, new representations are needed that can accommodate behaviors primitives with high variance.

## 6. Conclusion

In this paper, we describe *PICO*, an approach to learn behavior primitives from unlabeled demonstrations and a partial set of behavior primitives. We optimize a metric that directly minimizes reconstruction error for a set of demonstrations using sequences of behavior primitives. We directly compare our results to similar approaches using demonstrations generated from simulations of two different robotic platforms and achieve both better label accuracy and reconstruction accuracy as measured by action prediction mean squared error.

## Data availability statement

The original contributions presented in the study are included in the article/supplementary materials, further inquiries can be directed to the corresponding author/s.

## Author contributions

CR, KP, and CA carried out the experiments. All authors reviewed the manuscript.

## Conflict of interest

The authors declare that the research was conducted in the absence of any commercial or financial relationships that could be construed as a potential conflict of interest.

## Publisher's note

All claims expressed in this article are solely those of the authors and do not necessarily represent those of their affiliated organizations, or those of the publisher, the editors and the reviewers. Any product that may be evaluated in this article, or claim that may be made by its manufacturer, is not guaranteed or endorsed by the publisher.

## Footnotes

^1^ https://github.com/KyriacosShiarli/taco
